# Implementation research for the prevention of antimicrobial resistance and healthcare-associated infections; 2017 Geneva infection prevention and control (IPC)-think tank (part 1)

**DOI:** 10.1186/s13756-019-0527-1

**Published:** 2019-05-28

**Authors:** Walter Zingg, Julie Storr, Benjamin J. Park, Raheelah Ahmad, Carolyn Tarrant, Enrique Castro-Sanchez, Sara Tomczyk, Claire Kilpatrick, Benedetta Allegranzi, Denise Cardo, Didier Pittet, the 2017 Geneva IPC-Think Tank, Antoine Andremont, Mike Bell, Michael Borg, Yehuda Carmeli, John Conly, Philippe Eggimann, Petra Gastmeier, Lindsay Grayson, Stephan Harbarth, Marcela Hernandez, Loreen Herwaldt, Alison Holmes, John A. Jernigan, Amy Kolwaite, Karl-Heinz Krause, Elaine Larson, Sarah Masson-Roy, Shaheen Mehtar, Marc Mendelson, Ling Moi Lin, Andreea Moldovan, Dominique Monnet, Babacar Ndoye, Peter Nthumba, Folasade Ogunsola, Ben Park, Eli Perencevich, Matthew Samore, Wing Hong Seto, Arjun Srinivasan, Evelina Tacconelli, Maha Talaat, Maria Virginia Villegas, Andreas Voss, Tim Walsh, Andreas Widmer

**Affiliations:** 1 Infection Control Programme and WHO Collaborating Center, University of Geneva Hospitals and Faculty of Medicine, 4 Rue Gabrielle Perret-Gentil, 1211, 14 Geneva, Switzerland; 2 Infection Prevention and Control Global Unit, World Health Organization, Geneva, Switzerland; 3 US Centers for Disease Control and Prevention, Atlanta, GA USA; 4 National Institute for Health Research in Healthcare Associated Infection and Antimicrobial Resistance, Imperial College London, London, UK; 5 Department of Health Sciences, University of Leicester, Leicester, UK

**Keywords:** Implementation, Infection prevention and control, International, National, Institutional, Change, CDC, ECDC, WHO

## Abstract

**Background:**

Around 5–15% of all hospital patients worldwide suffer from healthcare-associated infections (HAIs), and years of excessive antimicrobial use in human and animal medicine have created emerging antimicrobial resistance (AMR). A considerable amount of evidence-based measures have been published to address these challenges, but the largest challenge seems to be their implementation.

**Methods:**

In June 2017, a total of 42 experts convened at the Geneva IPC-Think Tank to discuss four domains in implementation science: 1) teaching implementation skills; 2) fostering implementation of IPC and antimicrobial stewardship (AMS) by policy making; 3) national/international actions to foster implementation skills; and 4) translational research bridging social sciences and clinical research in infection prevention and control (IPC) and AMR.

**Results:**

Although neglected in the past, implementation skills have become a priority in IPC and AMS. They should now be part of any curriculum in health care, and IPC career paths should be created. Guidelines and policies should be aligned with each other and evidence-based, each document providing a section on implementing elements of IPC and AMS in patient care. International organisations should be advocates for IPC and AMS, framing them as patient safety issues and emphasizing the importance of implementation skills. Healthcare authorities at the national level should adopt a similar approach and provide legal frameworks, guidelines, and resources to allow better implementation of patient safety measures in IPC and AMS. Rather than repeating effectiveness studies in every setting, we should invest in methods to improve the implementation of evidence-based measures in different healthcare contexts. For this, we need to encourage and financially support collaborations between social sciences and clinical IPC research.

**Conclusions:**

Experts of the 2017 Geneva Think Tank on IPC and AMS, CDC, and WHO agreed that sustained efforts on implementation of IPC and AMS strategies are required at international, country, and hospital management levels, to provide an adequate multimodal framework that addresses (not exclusively) leadership, resources, education and training for implementing IPC and AMS. Future strategies can build on this agreement to make strategies on IPC and AMS more effective.

## Background

Around 5–15% of hospital patients worldwide suffer from healthcare-associated infections (HAIs) [1]. Years of excessive antimicrobial use in human and animal medicine have selected multidrug-resistant microorganisms, which have led to untreatable infections, often in vulnerable patient populations [2, 3]. This amounts to a global crisis that requires urgent actions in infection prevention and control (IPC) and antimicrobial stewardship (AMS) [4]. The European Centre for Disease Prevention and Control (ECDC) and the World Health Organization (WHO) have identified key/core components for effective organization of IPC at the facility (Table 1) and national levels (Table 2) [5–7]. Both IPC and combating antimicrobial resistance (AMR) are in national action plans in the Unites States [8], and other countries [9], and, WHO, together with the Food and Agriculture Organization (FAO) of the United Nations and the World Organisation for Animal Health (OIE), has issued a global action plan on AMR in 2015 that member states committed to adapt as national action plans [10, 11]. Thus, commitment at the highest national and international levels is already in place.

**Table 1:** Key and core components for effective infection prevention and control in health care facilities

	Key components (ECDC) [5]	Core components (WHO) [6]
1	An effective infection control program in an acute care hospital must include at least: one full-time specifically trained IPC nurse per 250 beds; a dedicated physician trained in IPC; microbiological support; data management support.	An IPC program with a dedicated, trained team should be in place in each acute healthcare facility for the purpose of preventing HAI and combating AMR through IPC good practices.
2	To make sure that the ward occupancy does not exceed the capacity for which it is designed and staffed; staffing and workload of frontline healthcare workers must be adapted to acuity of care; and the number of pool/agency staff minimized.	In order to reduce the risk HAI and the spread of AMR, the following should be addressed: 1) bed occupancy should not exceed the standard capacity of the facility; 2) healthcare worker staffing levels should be adequately assigned according to patient workload.
3	Sufficient availability of and easy access to material and equipment and optimized ergonomics.	At the facility level, patient care activities should be undertaken in a clean and/or hygienic environment that facilitates practices related to the prevention and control of HAI, as well as AMR; including all elements around the WASH infrastructure and services and the availability of appropriate IPC materials and equipment.
4	Use of guidelines in combination with practical education and training.	Evidence-based guidelines should be developed and implemented for the purpose of reducing HAI and AMR. Education and training of the relevant healthcare workers on guideline recommendations should be undertaken to achieve successful implementation.
5	Education and training involves frontline staff, and is team- and task-oriented.	At the facility level, IPC education should be in place for all healthcare workers by utilizing team- and task-based strategies that are participatory and include bedside and simulation training to reduce the risk of HAI and AMR.
6	Organizing audits as a standardized (scored) and systematic review of practice with timely feedback.	Regular monitoring/audit and timely feedback of healthcare practices should be undertaken according to IPC standards to prevent and control HAIs and AMR at the healthcare facility level. Feedback should be provided to all audited persons and relevant staff.
7	Participating in prospective surveillance and offering active feedback, preferably as part of a network.	Facility-based HAI surveillance should be performed to guide IPC interventions and detect outbreaks, including AMR surveillance with timely feedback of results to healthcare workers and stakeholders and through national networks.
8	Implementing IPC programs follows a multimodal strategy including tools such as bundles and checklists, developed by multidisciplinary teams and taking into account local conditions.	At the facility level, IPC activities should be implemented using multimodal strategies to improve practices and reduce HAI and AMR.
9	Identifying and engaging champions in the promotion of a multimodal intervention strategy.	NA
10	A positive organisational culture by fostering working relationships and communication across units and staff groups.	NA

*AMR*, Antimicrobial resistance; *ECDC*, European Centre for Disease Prevention and Control; *HAI*, Healthcare-associated infection; *IPC*, Infection prevention and control; *NA*, not applicable; *WASH*, Water, sanitation and hygiene; *WHO*, World Health Organization

**Table 2:** Core components for infection prevention and control at the national level

	National core components (WHO) [6]
1b	Active, standalone national IPC programs with clearly defined objectives, functions and activities should be established for the purpose of preventing HAI and combating AMR through IPC good practices. National IPC programmes should be linked with other relevant national programmes and professional organizations.
2	Evidence-based guidelines should be developed and implemented for the purpose of reducing HAI and AMR. Education and training of relevant healthcare workers on the guideline recommendations and the monitoring of adherence with guideline recommendations should be undertaken to achieve successful implementation.
3b	National IPC programs should support the education and training of the health workforce as one of its core functions.
4b	National HAI surveillance programs and networks including mechanisms for timely data feedback and with the potential to be used for benchmarking purposes should be established to reduce HAI and AMR.
5b	National IPC programs should coordinate and facilitate the implementation of IPC activities though multimodal strategies on a nationwide or sub-national level.
6b	National IPC monitoring and evaluation programs should be established to assess the extent to which standards are being met and activities are being performed according to the programs’ goals and objectives. Hand hygiene monitoring with feedback should be considered as a key performance indicator at the national level.

*AMR*, Antimicrobial resistance; *HAI*, Healthcare-associated infection; *IPC*, Infection prevention and control; *WHO*, World Health Organisation

Under the auspices of the US Centers for Disease Control and Prevention (CDC), the WHO, and the University of Geneva Hospitals and Faculty of Medicine (HUG), an international panel of 42 experts with backgrounds in IPC, microbiology, infectious diseases, public health, psychology, medical technology, and social sciences, convened for 2 days at the Geneva Think Tank on IPC and AMR in June 2017 (2017 Geneva IPC-Think Tank) to develop a vision on IPC and the prevention of AMR, and to agree on a road map for research and public health activities. Three dimensions on IPC and AMS were discussed: 1) implementation of IPC and AMS; 2) technology in IPC and AMS; and 3) broadening the global IPC network. This is the first in a series of three papers summarising the discussions at the 2017 Geneva IPC-Think Tank; it focuses on implementation of IPC and AMS.

## Methods

Four domains related to implementation of IPC and AMS were addressed: 1) teaching implementation skills to IPC professionals; 2) integrating implementation strategies in policy making on IPC and AMS; 3) national/international actions to support implementation of IPC and AMS; and 4) translational research bridging social sciences and research in IPC and AMS. After a presentation on the principles of implementation science and the ECDC IPC key components, and after a short introduction to the four domains to be discussed, the experts were allocated into four focus groups. The groups discussed each of the four domains for 30 min. The discussions were guided by a moderator, tape recorded, and writers took notes. The goal was to obtain a broad inventory of thoughts; the experts were invited to express their opinion and concerns towards the discussed domains. Summaries from the discussion rounds were shared in a plenary session.

## Results

Participants expressed both interest and general agreement with the principles of implementation research in IPC and AMS. The following paragraphs summarize aspects of implementation in IPC and AMS which were discussed in the discussion rounds, and examples, which were mentioned by the participants.

### Teaching “implementation” to IPC professionals

Implementation skills are important and should be integrated in the education of IPC and AMS. Flexible approaches to implementation informed by social science are the most valuable, in contrast to cookbook approaches or checklists, which risk being simplistic: fostering tick-boxing, rather than context specific, adapted, actions. HUG, to cite one example, has included training on “implementation” in its annual international course on implementation in IPC since 2014. The course follows a social sciences approach aiming at both sensitizing participants to the complexity of implementation research, and providing practical directions on tailoring implementation strategies in daily practice. The course is based on the “consolidated framework for implementation research” (CFIR) [12]. CFIR is a meta-theory, mapping several theories in implementation research to five dimensions: the intervention itself (to be adapted to the local context), the process of intervention (planning, engaging, executing, evaluating), the individuals involved in the intervention (personal beliefs, capacity, perceptions), the organizational context (structural and societal architecture), and determinants external to the organization (geography, culture, politics, healthcare system). The interplay of these dimensions is intuitive, and the practical explanations of the determinants given by the authors help IPC professionals to navigate through the dimensions.

Basic principles of implementation should be taught in undergraduate training, not only to IPC professionals, but to all healthcare professionals. Medical education could be supplemented to enable students to develop a more thorough understanding of social science and its contribution to knowledge about improvement and implementation. Training on implementation skills is a priority across low-and-middle-income (LMIC) and high-income countries. The need to accelerate discussion on the principles of implementation in IPC and AMS is not a luxury restricted to the “first” world nor is it a fad solely for researchers interested in social science. Implementation challenges that stop the translation of evidence into practice are real and apply everywhere. Education on any IPC or AMS activity should incorporate an implementation strategy. The IPC community should agree on the principles of implementation, and distinguish basic and advanced skills. Low-and-middle-income countries with limited access to skills platforms could benefit from e-learning resources provided or endorsed by international organizations and IPC societies. WHO has indeed made available, both as face-to-face training and as e-learning packages, a training module on “Leadership and programme management in infection prevention and control” which is deeply rooted in implementation science [13].

Also, social media could help promote knowledge. Particularly in LMICs, where smart phones can be found even in remote domains, social media may help in communication, education and training. Implementation (science) should become part of IPC certificates, as is the case already with the “European Certificate for Infection Control” (EUCIC) [14], established by the European Society for Clinical Microbiology and Infectious Diseases (ESCMID). EUCIC has integrated the HUG implementation course in its IPC curriculum. WHO or CDC could create a “model curriculum” for the implementation of strategies on IPC and AMS; in line with the “model legislation” it already proposes for Member States in some domains. Activities should be coordinated with multi-level partners, professional/civil societies and initiatives in related fields, e.g. antimicrobial resistance or water, sanitation and hygiene (WASH).

### Promoting implementation of strategies on IPC and AMS in policy making

Policies and guidelines on IPC and AMS are technical documents, issued by international organisations, national bodies and professional societies [15–19]. They are reference documents based on the evidence-base offering details on best clinical practice, and effective interventions and programmes. The term “implementation” is found in all of them, but as an advice (*do it*) rather than a concept (*how* to do it). WHO IPC guidelines and selected professional guidelines offer a perspective on “how” to implement recommendations [20, 21], but only a few have detailed sections on the subject [22–24]. Policies can support the implementation process on various levels: first, policies can offer practical information on implementation; second, policies can promote IPC and AMS as patient safety priorities; third, policies can define responsibilities and accountability surrounding best practice actions:

1. Each policy needs an implementation strategy with practical advice on project adoption (making it an organisational priority), project management, and education and training. When developing IPC guidelines, WHO develops solid implementation strategies and tools based on expert input from the field concurrently. The first, insightful example was its guideline on hand hygiene [22], accompanied by an innovative multimodal implementation strategy [25], and a number of practical tools to be used to facilitate recommendations’ adoption (http://www.who.int/infection-prevention/tools/hand-hygiene/en/). The intervention was pilot-tested in selected institutions worldwide, and tools were modified or adapted for optimal adoption of the change strategy [26]. Another example is the “Strategies to prevent central line-associated bloodstream infections in acute care hospitals” document issued by the Infectious Diseases Society of America (IDSA) in collaboration with the Society for Healthcare Epidemiology of America (SHEA), which included a section on implementation to their document [27].
2. Policies and guidelines should explain the purpose of the document, and how it is intended to be used within the health system [21]. They should discuss the magnitude of the problem and why it needs to be addressed [27]. Although IPC and AMS policies are about quality assurance, they promote “patient safety” actions. This is more than just a semantic detail; the term “safety” has more weight than “quality” and helps IPC professionals to get the attention of hospital managers, department leaders, and even the public. The strongest incentive to encourage hospitals to implement policies is when they become law, as has happened, for example, in Germany with the recommendations of the “Kommission für Krankenhaushygiene und Infektionsprävention” of the Robert Koch Institute [28].
3. Detailing and scope varies in guidelines, policies and recommendations. Some documents focus on best practice actions alone as if there was no context [15]. Others on the other hand, put best practice actions into a wider context addressing surrounding conditions such as accountability, non-technical interventions, and surveillance with timely feedback. Policies should define the responsibilities and accountability of the different stakeholders involved in IPC and AMS: from hospital manager to frontline worker [27]. Clear roles help IPC professionals communicate in the implementation process. Establishing surveillance with target setting (in combination with public reporting) enhances the value of a policy. Whether rewards or penalties (or both) are applied to achieve improvement depends on the national and organizational context (culture and law). Policies and guidelines have a role in bringing together AMS and IPC to avoid implementation problems that arise from treating them as separate topics.

Guidelines issued by international organisations, national bodies and professional societies are often not consistent [29]. Consistency and minimal standards set by international organisations such as CDC, ECDC or WHO would be helpful in this context. The key/core components published by ECDC and WHO already set the directions of successful IPC strategies [5, 6]. However, these strategies do not replace detailed policies, which need to be contextualised for each country. Ideally, national and local policies should be checked against international standards, contextually grounded, and provide tangible information about how to establish the recommendations. Not all countries, however, have the resources to build local guidelines: LMICs in particular tend to rely on international documents. Therefore, international organisations should aim to integrate the significance of local or regional contexts into the implementation sections. For example, WHO uses specific approaches to gather country and facility examples, including a focus on settings with limited resources, to develop its implementation manuals and resources [30, 31]. Allowing for adaptation is key to successful adoption by front line workers. If significant resources are required for a prevention strategy, policy makers should address this explicitly and discuss how implementation can be financed. In cases where urgent action would be required (e.g. outbreak situations), innovative resource allocation may be needed (e.g. ex-post reimbursement). Descriptions of implementation strategies should be packaged as structured, easy-to-read, protocols or manuals, and also published in the internationally available peer-reviewed literature. Ideally, guidelines, implementation strategies and tools for implementation should be translated into local languages.

### Activities at the national and international level

At the national level, authorities should be concerned about HAIs and AMR, and reposition IPC and AMS in national strategies. Indeed, this has been done in various countries in recent years. The role of policy makers at national level is not to enforce implementation as such, but to support implementation through political direction, legislation, and budget allocation. Today, IPC is perceived as a cost centre. This must change, and stakeholders need to accept the cost-benefit advantages of giving priority to patient safety. Political support helps IPC professionals in implementation at the institutional level. National IPC bodies should be established to coordinate activities with clearly defined tasks. Their work should involve different stakeholders, and they should be part of an international network. Clarifying the specific roles and responsibilities is critical. Health authorities or IPC bodies should promote awareness of IPC and AMS in the general public, by fostering patient engagement and encouraging media involvement. This can be achieved by using patient stories (giving patients a voice), or engaging marketing companies to promote prevention measures or judicious antimicrobial use.

On the international level, actions towards implementation are more about advocacy. There should be coordination and agreement on achievable aims and goals for IPC and AMS. Networking between countries should be encouraged. The activities of the ARHAI (antimicrobial resistance and healthcare-associated infection) network of ECDC and those of CDC and WHO in the context of the Global IPC Network are good examples. Leadership provided at international level, e.g. by CDC, ECDC, or WHO, helps to prioritise IPC and AMS within national budget discussions. In addition to education and training, for LMICs, it is necessary to establish funding mechanisms for materials delivered by international organisations. However, independent funding may face the challenge of sustainability. For both LMICs and high-income regions, the main task of international organisations is to prioritise and promote IPC and AMS. This sends a positive signal and facilitates implementation at institutional level. International ambassadors can further sensitize institutions and governments to the cause of IPC and AMS.

### Translational research

Evaluation of interventions can be challenging as in some instances, infections decrease already before the formal start of an intervention [32], or secular trends are stronger than the effect of a prevention programme [33]. There is strong evidence for the effectiveness of behavioural change interventions in IPC, whether they are bundled or non-bundled, and evidence indicates that implementation should follow a multimodal or multifaceted strategy [5, 34]. The challenge now is to learn more about the implementation of these types of intervention [35]. Requesting more detailed information on published interventions, and particularly about how interventions were implemented against organisational barriers would be valuable. The narrative of such detailing can help identify if a certain intervention is more or less likely to be replicated in a specific context. Over recent years, there has been growing collaboration between IPC professionals and social scientists to better understand the dynamics of implementing behavioural change interventions in IPC and AMS [32, 33, 36–39]. Methodologies used in qualitative social science research can help improve understanding. Ideally, mixed-methods studies, combining quantitative and qualitative research, should be promoted.

## Discussion

A perceived key challenge for IPC professionals today is the lack of skills in applying scientific evidence favouring specific healthcare practices in daily practice.

At the core of implementation research is the notion that scientific evidence is not sufficient to promote change in organisations [40]. This is because change is achieved by individuals within the organisations, who need to align new interventions and practices with their own education, beliefs and perceptions, and the context in which they work. Individuals are not passive recipients; they experiment with innovations, looking for meaning within them; they develop positive or negative feelings, and may challenge, worry or complain about any changes they may choose to work with some innovations while consciously or unconsciously avoiding others; or they may try to modify, improve or redesign an intervention – either on their own or by exchanging with other stakeholders [41]. Implementation will be shaped by organisational culture [42]: the array of norms, values, and basic assumptions shared by the “ensemble” of individuals. Like many other aspects of culture, organisational culture is stable, socially constructed, and partly subconscious. Also, characteristics of an institution and its social architecture (i.e. the people forming a group and the coordination of the group) are consequential: size, functional differentiation, resources all impact on implementation of strategies on IPC and AMS. The size and age of an organisation are both negatively associated with implementation success if bureaucratic structure is increased as a result. Power dynamics, social relations and team structures have been pinpointed as playing key roles in translating change into practice in healthcare settings and many different frameworks have been developed to structure the various forces that come into play when a potential change and an organisation come into contact with each other [12].

Given the complexity of organisations and their tendency to prefer the status quo, it is difficult for IPC professionals to implement IPC and AMS interventions that affect a range of employees of various levels and professions. In the absence of subtle tactics targeting behavioural change, implementation of best practice recommendations often follows two lines of argument: they are issued by a trusted authority, or they are issued with emphasis on the fact that they are evidence-based. The evidence base is, however, often ambiguous and contested and must be continually interpreted and reframed in accordance with priorities of the local context, a process that may involve power struggles among various professional groups [43]. Thus, implementation does not automatically follow evidence. Social scientists have contributed substantially to understanding implementation – indeed, implementation has become a science in its own right [12, 36, 41].

The 2017 Geneva Think Tank on IPC and AMS confirmed the importance of fostering implementation skills in IPC and AMS, and how little attention this had been given in the past. This is surprising because effective implementation of best practices in IPC and AMS is a critical part of delivering patient safety and quality. Best practices are effective, evidence-based procedures, performed by healthcare professionals in a given context. This context is influenced by hospital policy, organisational culture, resources, education and training, and perceptions. Stakeholders on many levels are accountable for patient safety, all within their domains of responsibility. Table 3 summarises actions to facilitate implementation in IPC and AMS, stratified by different stakeholder levels.

**Table 3:** Actions to facilitate implementation in infection prevention and control and antimicrobial stewardship

Level	Action
International level	- Make IPC a global priority – take the lead - Support global IPC initiatives - Provide minimal standards in patient safety - Provide guidance (key/core components)
National level	- Make IPC a national priority – have a national strategy - Provide career paths for IPC professionals - Promote IPC and AMS, as well as their implementation, being part of education and training for all healthcare professionals - Provide national guidelines and policies, aligned with international standards - Organise surveillance of process and outcome indicators, benchmark with other countries - Make resources available (education and training, materials, staffing)
Health care facility level	- Make IPC an institutional priority – have a local strategy - Provide resources - Allow postgraduate training in IPC and AMS as well as their implementation
IPC community	- Integrate implementation science, management, and leadership in the curriculum of IPC training - Make implementation science an ongoing topic on the agenda of any IPC workshop
Research	- Add an implementation narrative to any publication on best practice interventions - Encourage collaboration between IPC researchers and social scientists to improve study design and reporting - Provide funding of mixed-methods research on behaviour change interventions

*AMS*, Antimicrobial stewardship; *IPC*, Infection prevention and control

Countries should adopt an implementation-focused attitude, making the successful implementation of IPC and AMS a national priority, collaborate with other countries or international organisations, assure appropriate education and training, make IPC a respected career path, provide sufficient resources, and produce policies that can be implemented at the various levels of health delivery. The situation in LMICs is dominated by a lack of resources and infrastructures and organisational needs that are not being met.

International organisations should make HAI-prevention and AMS a priority. They also have a role to play by working with IPC and water, sanitation and hygiene (WASH) professionals and institutions in LMICs through freely accessible education and training modules and the development, co-development and provision of practical, contextually relevant implementation tools. Research in IPC and AMR should embrace qualitative research. Studies, most often observational, should have an implementation narrative, the story around barriers and facilitators and what needs to be considered in order to succeed. This would help to adapt prevention strategies to the local context or to decide if a certain IPC or AMS implementation strategy is suitable at all. Adaptation is key to better adoption of IPC/AMS implementation at the patient bedside. We need to collaborate with social scientists to improve the way prevention strategies are designed, implemented and reported. Advocacy should be done to convince donors about the importance of research funding for qualitative research or mixed-methods studies.

## Conclusion

Experts of the 2017 Geneva Think Tank on IPC and AMS, CDC, and WHO agreed that sustained efforts on implementation of strategies on IPC and AMS is required at international, country, and hospital management levels to provide an adequate multimodal framework that addresses (not exclusively) leadership, resources, education and training for implementing IPC and AMS. Future strategies can build on this agreement to make strategies on IPC and AMS more effective.

## Abbreviations

AMR: Antimicrobial resistance
AMS: Antimicrobial stewardship
CDC: US Centers for Disease Control and Prevention
CFIR: Consolidated Framework for Implementation Research
ECDC: European Centre for Disease Prevention and Control
ESCMID: European Society for Clinical Microbiology and Infectious Diseases
EUCIC: European Certificate for Infection Control
HAI: Healthcare-associated infection
HUG: University of Geneva Hospitals
IDSA: Infectious Diseases Society of America
IPC: Infection prevention and control
LMIC: Low-and-middle-income country
SHEA: Society for Healthcare Epidemiology of America
WASH: Water, sanitation and hygiene
WHO: World Health Organization
